# High phosphate reduces host ability to develop arbuscular mycorrhizal symbiosis without affecting root calcium spiking responses to the fungus

**DOI:** 10.3389/fpls.2013.00426

**Published:** 2013-10-29

**Authors:** Coline Balzergue, Mireille Chabaud, David G. Barker, Guillaume Bécard, Soizic F. Rochange

**Affiliations:** ^1^Laboratoire de Recherche en Sciences Végétales, Université de Toulouse, Université Paul Sabatier, UMR5546Castanet-Tolosan, France; ^2^Centre National de la Recherche Scientifique, UMR5546Castanet-Tolosan, France; ^3^Laboratory of Plant–Microbe Interactions, Institut National de la Recherche Agronomique (UMR441), Centre National de la Recherche Scientifique (UMR2594)Castanet-Tolosan, France

**Keywords:** mycorrhiza, phosphate, symbiosis, molecular signaling, calcium spiking, chito-oligosaccharides

## Abstract

The arbuscular mycorrhizal symbiosis associates soil fungi with the roots of the majority of plants species and represents a major source of soil phosphorus acquisition. Mycorrhizal interactions begin with an exchange of molecular signals between the two partners. A root signaling pathway is recruited, for which the perception of fungal signals triggers oscillations of intracellular calcium concentration. High phosphate availability is known to inhibit the establishment and/or persistence of this symbiosis, thereby favoring the direct, non-symbiotic uptake of phosphorus by the root system. In this study, *Medicago truncatula* plants were used to investigate the effects of phosphate supply on the early stages of the interaction. When plants were supplied with high phosphate fungal attachment to the roots was drastically reduced. An experimental system was designed to individually study the effects of phosphate supply on the fungus, on the roots, and on root exudates. These experiments revealed that the most important effects of high phosphate supply were on the roots themselves, which became unable to host mycorrhizal fungi even when these had been appropriately stimulated. The ability of the roots to perceive their fungal partner was then investigated by monitoring nuclear calcium spiking in response to fungal signals. This response did not appear to be affected by high phosphate supply. In conclusion, high levels of phosphate predominantly impact the plant host, but apparently not in its ability to perceive the fungal partner.

## INTRODUCTION

As an essential component of many biomolecules such as nucleic acids, proteins, and membrane phospholipids, phosphorus (P) plays an essential role in the structure and physiology of all living cells. In plants, P availability is considered the second most important limiting factor for growth after nitrogen. While P is generally abundant in soil, it is mostly present in insoluble and poorly mobile forms and therefore partly unavailable to plants (Schachtman et al., 1998). Roots take up P as inorganic phosphate (Pi), and this leads to the creation of Pi depletion zones around them, a phenomenon that can lead to P deprivation. Crop plants are thus commonly supplied with chemical P fertilizers, which raises several major economic and environmental concerns related to energy use, freshwater pollution, and mineral P resource scarcity (Cordell et al., 2009; Gilbert, 2009).

In addition to several mechanisms of internal P remobilization (Plaxton and Tran, 2011), plants facing P deprivation display a number of adaptive responses that enhance their P uptake capacity. The means by which roots can acquire Pi from the soil can be classified into two main pathways (Smith and Smith, 2011). The direct uptake pathway, present in all plants, involves the activity of root Pi transporters. The efficiency of this pathway can be enhanced through the solubilization of chelated soil P by secreted organic acids and enzymes, the expression of high-affinity Pi transporters (Poirier and Bucher, 2002; Grunwald et al., 2009), or changes in root system architecture that provide access to a larger volume of soil (Péret et al., 2011). Some of these adaptations occur in response to local Pi availability (Svistoonoff et al., 2007), while others are regulated at the systemic level as a function of the plant P nutritional status (Thibaud et al., 2010).

The majority of plant species possess an additional P uptake route called the symbiotic pathway. This involves an intimate connection between roots and soil fungi. Among such organisms, arbuscular mycorrhizal (AM) fungi interact with the largest number of plant partners (approximately 80% of plant species; Smith and Read, 2008), forming the most widespread symbiosis on earth (Brachmann and Parniske, 2006). In this root endosymbiosis, the fungal partner colonizes the root cortex where it forms specialized structures called arbuscules that serve as an exchange interface. At the same time, the fungus develops a dense hyphal network that extends far into the soil while still remaining connected to the root. This extraradical mycelium provides the plant with water and nutrients that would otherwise remain inaccessible to roots. Among supplied nutrients, Pi is considered as quantitatively the most important (Smith and Read, 2008). AM fungi can obtain free Pi from the soil using high-affinity Pi transporters expressed in the mycelium (Harrison and van Buuren, 1995). Once taken up by the extraradical mycelium, P is translocated along the hyphae in the form of polyphosphates, which are then depolymerized so that Pi can be transferred to root cells in exchange for hexoses (Ohtomo and Saito, 2005). This last step involves both plant and fungal transporters located at the periphery of arbuscules (Bapaume and Reinhardt, 2012). In some cases the symbiotic pathway can account for the entire P uptake, as demonstrated by the use of radiolabeled P made available only to the fungus (Smith et al., 2004; Smith and Smith, 2011).

Prior to contact, AM fungi and their host roots release molecular factors into the rhizosphere. Certain features of host–fungal signaling are similar to those described for the nitrogen-fixing symbiosis that associates rhizobia with legumes, and in particular the essential role of several plant genes comprising the so-called “common signaling pathway” (Singh and Parniske, 2012). A hallmark of this signaling pathway is the induction of peri- and intra-nuclear oscillations of calcium concentration (known as calcium spiking) in response to microbial compounds (Ehrhardt et al., 1996; Kosuta et al., 2008; Chabaud et al., 2011). This calcium signal is likely decoded by a calcium- and calmodulin-dependent kinase, leading to the activation of appropriate transcription factors and downstream genes necessary for the establishment of the functional interaction.

In the case of mycorrhizal interactions, early molecular signals exchanged between the symbionts were identified only recently. Plant roots release strigolactones into the rhizosphere that can stimulate hyphal branching and respiratory metabolism of AM fungi (Akiyama et al., 2005; Besserer et al., 2006, 2008). Through the analysis of pea mutants defective in strigolactone biosynthesis, these compounds were found to be important for a normal level of mycorrhizal root colonization (Gomez-Roldan et al., 2008), although symbiotic structures appeared morphologically unaltered in the mutants. A similar phenotype of reduced mycorrhization was observed in *Petunia* mutants defective for the strigolactone exporter PhPDR1 (Kretzschmar et al., 2012), which demonstrated that strigolactone transport is essential for the function of these signals in AM symbiosis. These studies suggest an important role for strigolactones in the stimulation of the fungus outside the roots, and possibly also in the progression of AM fungal hyphae within roots.

Reciprocally, AM fungi release compounds that trigger a variety of responses in plant roots, including calcium spiking, changes in gene expression and lateral root formation (Parniske, 2008). Two classes of such compounds were identified recently, both comprising an *N*-acetylglucosamine oligomer backbone. Firstly, lipochitooligosaccharides called Myc-LCOs, structurally similar to the Nod factors that mediate the nitrogen-fixing symbiosis, are able to stimulate lateral root formation and the colonization of roots by AM fungi (Maillet et al., 2011). Secondly, short-chain chitooligosaccharides (Myc-COs) can trigger nuclear calcium spiking in host plant root cells and their concentrations in fungal exudates are stimulated by strigolactones (Genre et al., 2013).

The establishment of the AM symbiosis can be disturbed by environmental conditions, including P availability which can inhibit the symbiotic interaction (e.g., Menge et al., 1978; Thomson et al., 1986; Breuillin et al., 2010; Bonneau et al., 2013). This is often interpreted as a means for plants to avoid the carbon cost of symbiosis (up to 20% of photosynthetic carbon can be directed to AM fungi; Bago et al., 2000) when sufficient Pi can be acquired through the direct uptake pathway (Nagy et al., 2009). Nonetheless, this regulation also deprives the plant of other benefits of AM symbiosis, including improved water uptake, nitrogen supply, and enhanced resistance to pathogens (Smith and Read, 2008). Interestingly, recent studies have demonstrated a cross-talk between Pi and nitrogen availabilities to control AM associations (Javot et al., 2011; Bonneau et al., 2013), thus indicating a high level of integration of mycorrhizal responses to mineral nutrition.

Depending on the experimental system, both the extent to which and the stage when the symbiosis is inhibited by P can differ markedly (Gosling et al., 2013). This suggests the existence of multiple regulatory mechanisms that can either prevent the establishment of the symbiosis in the first place or lead to the elimination of the AM fungus from roots after it has engaged in a functional interaction (Koide and Schreiner, 1992). Some authors have reported a direct effect of P on AM spore germination and hyphal growth (de Miranda and Harris, 1994), while others were unable to detect any effect of P supply on these presymbiotic events (e.g., Schwab et al., 1983; Balzergue et al., 2011). In some cases indirect effects of P on the fungus through an alteration of root exudate content have been demonstrated (Nair et al., 1991; Tawaraya et al., 1998). Evidence has also been gathered for downregulation by P of well established AM interactions, via a reduced production of root compounds (Akiyama et al., 2002). The diversity of these observations suggests that multiple layers of control exist (Breuillin et al., 2010) and that the predominent regulatory mechanisms depend to a large extent on the plant and fungal species under study, as well as on the co-culture conditions and mode of P supply.

In a previous study carried out with pea, we found that the AM symbiosis could be arrested almost completely by a high P supply at a very early stage, prior to the attachment of the fungus on the root (Balzergue et al., 2011). We also confirmed previous reports (Yoneyama et al., 2007; López-Ráez et al., 2008) that the synthesis and exudation of strigolactones are negatively affected by a high availability of Pi, and showed that this effect, like the inhibition of AM symbiosis, is regulated at the systemic level (Balzergue et al., 2011). These observations revealed strigolactones as good candidates for mediating the effect of Pi on AM symbiosis. Under conditions of P sufficiency, a strong reduction of strigolactone release would prevent the stimulation of AM fungi and hence the establishment of the interaction. However, an exogenous supply of strigolactones was unable to restore mycorrhization under high P conditions, indicating that reduced strigolactone production was not the sole explanation for the absence of mycorrhizae (Breuillin et al., 2010; Balzergue et al., 2011). Therefore, additional mechanisms targeting the early steps of AM symbiosis establishment remain to be discovered.

The aim of the present study was to investigate how the AM symbiosis is inhibited by P in the model legume *Medicago truncatula*, with a particular focus on early stages of the interaction. We attempt to determine whether a high P supply primarily targets the plant or fungal partner and investigate the plant nuclear calcium spiking response to the fungus or fungal signals.

## MATERIALS AND METHODS

### BIOLOGICAL MATERIALS AND GROWTH CONDITIONS

Seeds of* M. truncatula* Gaertn genotype Jemalong A17 were scarified for 7 min in concentrated sulfuric acid and rinsed several times with sterile water. Seeds were then surface-sterilized in 2.6% sodium hypochlorite for 2 min and rinsed five times with sterile water. Seeds were transferred to water-agar plates [0.8% (w/v)] for 5 days at 4°C in the dark, then for 24 h at 25°C (16 h photoperiod). Germinated seedlings were transferred to pots containing 150 mL of sterilized charred clay (Oil-Dri, Brenntag, France) as a substrate. Plants were placed in a growth chamber with a 16 h photoperiod (22°C day, 20°C night). They were fertilized daily with half-strength Long Ashton nutrient solution (Hewitt, 1966) containing a final concentration of either 0.0075 mM (low P) or 3.75 mM (high P) sodium dihydrogen phosphate.

*Medicago truncatula* root organ cultures expressing the 35S:NupYC2.1 construct (Sieberer et al., 2009) were obtained as described by Chabaud et al. (2011) and grown in vertical Petri dishes to favor a regular fishbone-shaped root system (Chabaud et al., 2002). Transgenic *M. truncatula* plants expressing the 35S:NupYC2.1 construct were obtained by *Agrobacterium tumefaciens *transformation**(Genre et al., 2013). T1 and T2 lines expressing the transgene were selected for use in this study.

Sterile spores of *Rhizophagus irregularis* (DAOM 197198, formerly *Glomus intraradices*; Krüger et al., 2012) were purchased from Agronutrition (Labège, France). Spores of *Gigaspora gigantea* (isolate HC/F E30, Herbarium Cryptogamicum Fungi, University of Torino, Italy) were produced and sterilized as described in Besserer et al. (2006).

### PLANT INOCULATION AND DETERMINATION OF MYCORRHIZAL RATE

Plants were inoculated with 90 spores of *R. irregularis* per pot. Sixty spores were mixed with the substrate and 30 were added close to the seedling. The percentage of root length colonized by the fungus (i.e., showing arbuscules, vesicles, or both) was determined by the gridline intersection method (Giovannetti and Mosse, 1980), using a dissecting microscope after sampling of root fragments and staining with Schaeffer black ink (Vierheilig et al., 1998).

### DETERMINATION OF PHOSPHATE CONTENT

Leaf or root tissue samples were ground in 10% (w:v) perchloric acid using a FastPRep system with lysing matrix A (MP Biomedicals). Inorganic phosphate content in the supernatant was determined by the colorimetric method based on molybdenum blue described in Nanamori et al. (2004). Briefly, absorbance at 820 nm was measured after incubation of supernatant samples with ammonium molybdate in the presence of sulfuric acid and ascorbic acid.

### GENE EXPRESSION ANALYSIS

Gene expression analysis was carried out by reverse transcription-quantitative PCR (RT-qPCR) as part of a Dynamic Array^TM^ integrated fluidic circuits experiment, using a 96.96 Dynamic Genotyping chip (Fluidigm, BMK-M-96.96GT).

Non-inoculated *M. truncatula* plants were grown for 2 weeks (16 h photoperiod, 70% humidity) and fertilized with low P or high P nutrient solution. For each condition, the entire root systems of four plants were pooled and ground in liquid nitrogen. Extraction of total RNA was performed using the RNeasy plant mini kit (Qiagen) according to the manufacturer’s protocol. The RNA concentration was determined with a Nano Drop® ND-1000 and RNA quality was estimated using an Agilent RNA 6000 nano series II chip prior to DNase treatment (Ambion® TURBO DNA-free). One microgram of RNA was reverse-transcribed using SuperScript^TM^ III reverse transcriptase (Invitrogen). cDNA samples were diluted to a concentration of 60 ng/μL and subjected to pre-amplification (TaqMan® PreAmp kit). For each condition, three independent biological replicates were performed and each sample was analyzed in technical duplicate.

Primers used for qPCR are listed in **Table 1**. Real qPCR efficiencies were calculated using LinRegPCR software (Ramakers et al., 2003) for each primer pair (the average efficiency was calculated for all reactions using this primer pair). The expression of the genes of interest was calculated relative to four reference genes (geometric mean of *MtEF1α*, *MtHLC*, *MtPDF2*, and *MtPPRep*) taking into account the real PCR efficiency for each primer pair (Pfaffl, 2001).

**Table 1:** Oligonucleotide sequences

*EF1α*	Forward	CTTTGCTTGGTGCTGTTTAGATGG	Maillet et al. (2011)
	Reverse	ATTCCAAAGGCGGCTGCATA	
*HLC*	Forward	GTACGAGGTCGGTGCTCTTGA	Mbengue et al. (2010)
	Reverse	GCAACCGAAAATTGCACCATA	
*PDF2*	Forward	GTGTTTTGCTTCCGCCGTT	Kakar et al. (2008)
	Reverse	CCAAATCTTGCTCCCTCATCTG	
*PPRep*	Forward	GGAAAACTGGAGGATGCACGTA	Kakar et al. (2008)
	Reverse	ACAAGCCCTCGACACAAAACC	
*PT1*	Forward	GGGATGTTATGCACATTACTT	Liu et al. (2008)
	Reverse	CCAGTAGCTAAATGCAAACAG	
*PT2*	Forward	GGGATGTTATGCACATTACTT	Liu et al. (2008)
	Reverse	CCTATGGAGTGGAAAAATAGA	
*PT3*	Forward	TTCAGCAAGCAATTCGCAAAACG	Grunwald et al. (2009)
	Reverse	GTGAACCAGTAGCCCGGAACAGTA	
*PT5*	Forward	CTGAGTATGCGAACAAGAAGA	Grunwald et al. (2009)
	Reverse	ACGCCAGTAATAGGTAAGTGC	
*PHR1*	Forward	ATCACTCACGCGCTGCGATG	Branscheid et al. (2010)
	Reverse	AACGGCAACGAACGGATGCG	
*PHO2*	Forward	GGAGCCTCCACAGTTCTTCAAG	Branscheid et al. (2010)
	Reverse	AAGGACAAGAGCCTGCAGAGAG	
*Mt4*	Forward	AATGATTGCTGGGAATGAACCTT	Branscheid et al. (2010)
	Reverse	TTCCAAAGAGAAAATCCCATCAA	
*Spx*	Forward	CAGGATAGGTGCTGAGTTTAGCTCT	Branscheid et al. (2010)
	Reverse	GAGAAACAGGAAACACGCGAA	
*D27*	Forward	GAGATGATATTCGGCCAGGAAC	Liu et al. (2011a)
	Reverse	GCATGGTTTTTCTTAGCCTTGC	
*CCD7*	Forward	CCAAACAAACCTGAAAGCAA	
	Reverse	ATTTCCAAATTCCCATGAGC	
*CCD8*	Forward	ACTACAACTTCAGGCACCTC	
	Reverse	GAGATTCAACTTGCCGATGG	
*MAX1*	Forward	TTGGGTTTGGTTAGCCCTTG	Liu et al. (2011a)
	Reverse	CGCAGTTAGGGTCAAACCTTTC	

*Primers used for qRT-PCR to quantify primary transcripts of reference genes (*EF1α*, *HLC*, *PDF2*, *PPRep*), phosphate related genes (*PT1*, *PT2*, *PT3*, *PT4*, *PT5*, *PHR1*, *PHO2*, *Mt4*, *Spx*), and strigolactone biosynthesis related genes (*D27*, *CCD7*, *CCD8*, *MAX1*). The last column indicates the articles in which these sequences were originally published, where applicable. EF1α, translation elongation factor 1α; HLC, helicase; PDF2, rotodermal factor2; PPRep, pentatricopeptide repeat protein; PT, phosphate transporter; PHR1, phosphate starvation response 1; PHO2, phosphate2; D27, Dwarf27; CCD, carotenoid cleavage dioxygenase; MAX1, more axillary growth1.*

### INOCULATION SYSTEM TO INDEPENDENTLY CONTROL HOST AND FUNGAL P STATUS

Root exudates of *M. truncatula* were produced as follows. Plants were grown for 3 weeks and fertilized with low P or high P nutrient solution. The plants were gently uprooted and the roots still attached to the shoot were carefully freed from the substrate, rinsed, and then exudates were produced by immersion of roots in 200 mL of the same nutrient solution for 24 h. A fresh batch of root exudates was produced for each treatment of spores, and filter-sterilized before use.

Replicates of 500 sterile spores of *R. irregularis* were put into 40 μm cellular sieves (BD Falcon^TM^) placed in 6-well plates (Nunc). Eight milliliters of sterile root exudates were added to each batch of spores. Plates were incubated at 30°C in the dark under 2% CO_2_ for a total period of 15 days during which three treatments with root exudates were performed. For the second and third treatments, sieves containing spores were transferred to fresh plates prior to addition of fresh root exudates.

In parallel, *M. truncatula* plants were grown for 10 days in 15-mL plastic cylinders, the bottom of which was closed by a piece of nylon membrane. To place the roots of these plants in contact with the AM fungus, the membrane was removed and each cylinder was placed on a cellular sieve containing stimulated spores. Assembled systems were placed in charred clay substrate watered with low P or high P nutrient solution to ensure sufficient moisture for 5 days. At the end of the experiments, whole root systems were stained and observed as described above for the assessment of AM root colonization.

### NUCLEAR CALCIUM SPIKING ANALYSES

Oscillations of nuclear calcium concentration were monitored using the NupYC2.1 calcium sensor (Watahiki et al., 2004) driven by the cauliflower mosaic virus 35S promoter (Sieberer et al., 2009). The cameleon sensor protein YC2.1 undergoes a conformational change when bound to calcium, which leads to a change in the YFP to CFP ratio by Förster resonance energy transfer (FRET). Compared to other calcium sensors, cameleon proteins offer the advantages of being addressed to a particular cell compartment, as well as sensitive detection at the single-cell level. NupYC2.1 corresponds to a translational fusion of YC2.1 with the nuclear protein nucleoplasmin, ensuring nuclear localization of the sensor.

Root organ cultures or whole plants were grown on M medium (Bécard and Fortin, 1988) containing 0.035 mM (low P) or 3.5 mM (high P). For inoculation *G. gigantea* spores were pregerminated on either low P or high P M medium at 30°C in the dark under 2% CO_2_. Three days later, germinated spores were transferred to corresponding low P or high P plates containing *M. truncatula* root organ cultures expressing the 35S:NupYC2.1 construct. Roots and fungi were covered with Biofolie 25^TM^ (Dutscher SAS, Brumath, France) as described in Genre et al. (2005). After 15 days of co-culture, zones containing highly branched hyphae were visually selected to search for epidermal cells contacted by hyphopodia. These cells, as well as underlying cells, were directly analyzed using a confocal laser-scanning microscope as described in Chabaud et al. (2011). Calcium spiking was recorded over 10 min under each hyphopodium analyzed. In parallel, root samples were taken from other zones containing highly branched hyphae, in order to assess the frequency of hyphopodium formation in low P and high P. These roots were stained and examined as detailed above for the presence of mycorrhizal structures.

For the analysis of calcium spiking in response to fungal signals, young lateral roots excised from root organ cultures or whole transgenic plants were placed in a microchamber. One hundred microlitre of treatment solution was applied to the roots immediately prior to analysis under the confocal microscope. Negative controls were performed on the same root explants that were used for treatment. Explants incubated in water were analyzed for 10–15 min prior to treatment. No calcium spiking could be detected in water, except in rare cases (<3% of nuclei) where one isolated spike was observed. Explants were then treated by CO4 or germinated spore exudates (GSEs), and spiking was always observed within the first 10 min following treatment. For each treatment several roots were tested, and for each root 10–15 nuclei were observed. Solutions used for treatment were either 10^-8^ M CO4 or *R. irregularis* GSEs obtained as described in Genre et al. (2013). YFP and CFP fluorescence intensities were recorded over 30 min and data were processed as described in Genre et al. (2013).

### STATISTICAL ANALYSES

Statistical analyses were performed using SigmaStat or Statgraphics Centurion XV.II professional software packages. Data sets that satisfied normality and homoscedasticity criteria were compared using the Student’s *t*-test or analysis of variance (ANOVA) followed by the Fisher’s least significant difference (LSD) tests. The unequal variance *t*-test (Welch’s test) was used for two-sample comparisons when data fitted a normal distribution but variances were unequal. The Mann–Whitney’s rank sum test was used when data did not fit a normal distribution.

## RESULTS

### CHOICE OF P FERTILIZATION REGIMES

**FIGURE 1:**
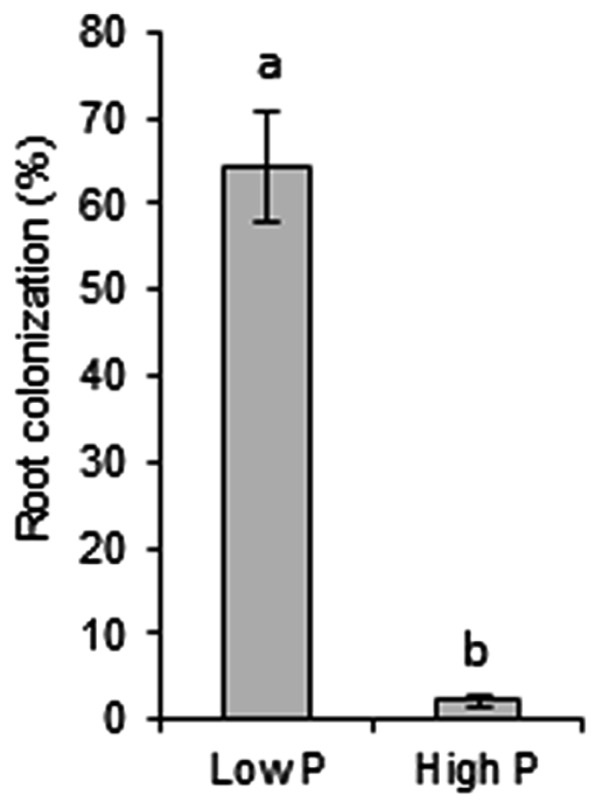
**Effect of P fertilization on AM root colonization of *M. truncatula.*** Plants were inoculated with spores of *R. irregularis* and fertilized with low P or high P nutrient solutions containing 0.0075 or 3.75 mM Pi, respectively. The extent of root colonization was determined 5 weeks post-inoculation after observation of stained root samples, and is shown as the fraction of the root length with arbuscules, vesicles, or both. Error bars represent SEM. *n* = 6 plants per condition. Different letters indicate a statistically significant difference according to the unequal variance *t*-test (*P* < 0.001).

**FIGURE 2:**
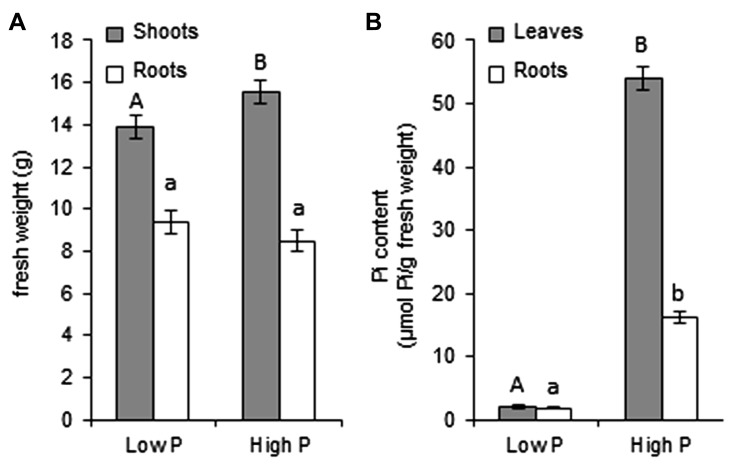
**Effect of P supply on growth and internal Pi content**. Plants were grown for 5 weeks with low P or high P fertilization. Error bars represent the SEM. **(A)** Shoot (gray bars) and root (white bars) fresh weights were determined; *n* = 5 plants per condition. Different letters indicate a statistically significant difference according to the Student’s *t*-test (shoots: upper case, *P* = 0.045; roots: lower case, *P* = 0.223). **(B)** Free Pi content was measured in extracts from leaves (gray bars) and roots (white bars); *n* = 3 plants per condition. Different letters indicate statistically significant differences according to the Student’s *t*-test (leaves: upper case, *P* < 0.001; roots: lower case, *P* < 0.001).

In order to study signaling events involved in the establishment of the AM symbiosis in *M. truncatula*, we first needed to determine the experimental conditions necessary to obtain a clear P-dependent phenotype. Plants were inoculated with spores of the AM fungus *R. irregularis* and fertilized with nutrient solutions containing different concentrations of Pi. The percentage of the root length colonized by the fungus (i.e., showing arbuscules, vesicles, or both) was determined after 5 weeks of co-culture. The Pi concentration that had been used to inhibit mycorrhizal colonization in pea (0.75 mM; Balzergue et al., 2011) was not adequate for *M. truncatula *(not shown)*.* A Pi concentration of 3.75 mM was sufficient to almost completely block mycorrhization, with root colonization remaining under 2% [vs 62% at 0.0075 mM Pi (**Figure 1**)]. The two fertilization regimes of 0.0075 and 3.75 mM Pi will subsequently be referred to as low P and high P, respectively. It is worth noting that although very few colonization events were observed under high P, whenever the fungus successfully entered the roots the subsequent steps of the interaction appeared to proceed normally and arbuscules did not display any morphological abnormalities. Biomass and Pi content were determined under these two contrasting fertilization conditions. High P supply hardly affected plant growth: shoot biomass was significantly but only moderately increased while root biomass remained unchanged (**Figure 2**). Total biomass was unaffected by P supply (Student’s *t*-test, *P* = 0.606). In contrast high P conditions strongly enhanced Pi content in leaves, and to a lesser extent in roots (**Figure 2**).

**FIGURE 3:**
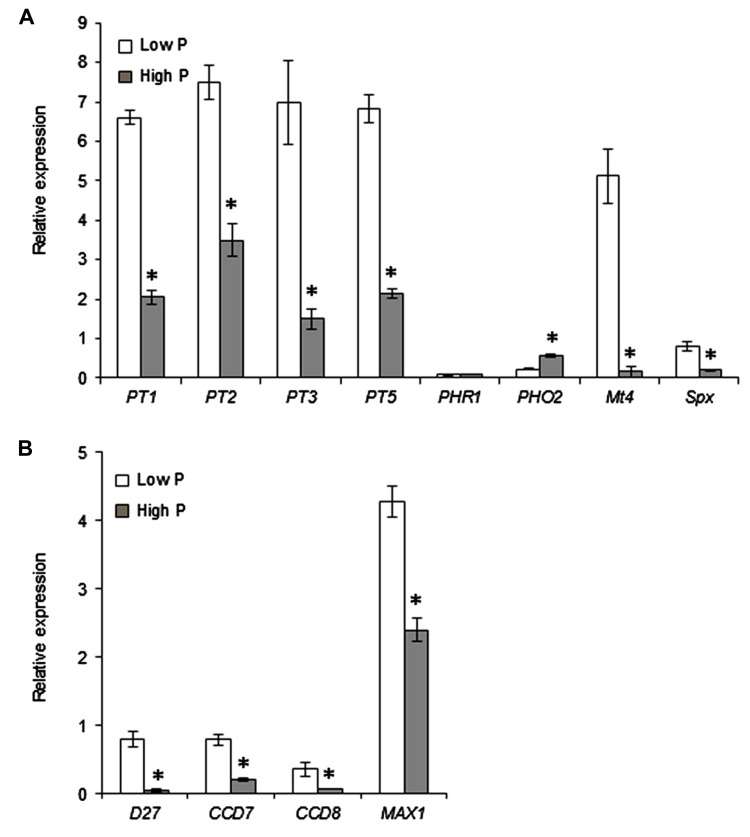
**Effect of Pi supply on the expression of selected genes**. Expression levels were determined relative to constitutive reference genes. Error bars represent the SEM; *n* = 3 biological replicates per condition. Asterisks indicate a statistically significant difference between low P and high P (*P* < 0.05) for each gene, according to the Student’s *t*-test or to the unequal variance *t*-test where appropriate. **(A)** Genes involved in Pi acquisition (root Pi transporters: *MtPT1*, *MtPT2, MtPT3, MtPT5*) and markers of Pi starvation (*MtPHO2*, *MtPHR1*, *Mt4*, *MtSpx*). **(B)** Strigolactone biosynthetic genes.

To further investigate the nutritional status of plants grown under low P and high P, and validate the contrasting conditions of P supply, the expression of a set of marker genes was examined. Phosphate transporter genes known to be regulated by P supply, such as *MtPT1*, *MtPT2*, *MtPT3*, and *MtPT5* (Liu et al., 2008; Grunwald et al., 2009) were found to be 2.2- to 4.7-fold more highly expressed under low P (**Figure 3A**). The expression levels of other genes related to P starvation signaling (*Mt4* and *MtSpx*; Burleigh and Harrison, 1997; Duan et al., 2008) were also respectively 31.9- and 3.8-fold higher under low P. The upregulation of *Mt4* in low P was particularly strong, consistent with the Northern blot analysis reported by Burleigh and Harrison (1998). In contrast *MtPHO2*, a negative regulator of P starvation responses (Delhaize and Randall, 1995; Bari et al., 2006) was more highly expressed under high P. Another important regulator of Pi starvation responses is PHR1 (Rubio et al., 2001), a transcription factor known to be regulated at the post-translational level (Miura et al., 2005). As expected, the expression of *MtPHR1* was unaffected by P (**Figure 3A**).

We next set out to determine whether the P supply conditions affect the biosynthesis of the important root-derived signals strigolactones. Because strigolactones are produced in very low quantities, their biochemical detection is difficult in many species, and the expression of biosynthetic genes is commonly used as an indirect assessment of strigolactone production (Vogel et al., 2010). We found that the expression of four genes involved in strigolactone biosynthesis, *MtD27*, *MtCCD7*, *MtCCD8*, and *MtMAX1*, were downregulated under high P (**Figure 3B**). This effect was most important for *MtD27*, which encodes the first enzyme in the strigolactone biosynthetic pathway (Alder et al., 2012), indicating a reduced synthesis of strigolactones under high P conditions.

### INOCULATION SYSTEM TO INDEPENDENTLY CONTROL HOST AND FUNGAL P STATUS

**FIGURE 4:**
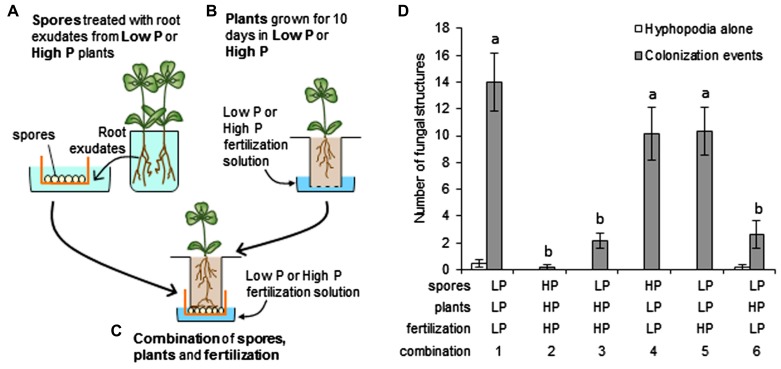
**Inoculation system to independently control host and fungal P status**. **(A–C)** Steps in the experimental design. **(A)** *Medicago truncatula *plants**grown in low P or high P**produced root exudates used to stimulate *R. irregularis *spores placed in cellular sieves. Three treatments with root exudates were applied over a total of 15 days. **(B)** Another set of *M. truncatula *seedlings were grown in small plastic containers closed by a mesh (dotted line) for 10 days in low P or high P. **(C)** The mesh was removed from the containers used in **(B)** and the seedlings placed inside the sieves containing stimulated spores (obtained in **(A)**), so that the roots were in direct contact with the spores. Assembled units were watered with low P or high P nutrient solution for 5 days. **(D)** Mycorrhizal structures were examined on whole root systems of each plant. Root zones displaying hyphopodia alone (open bars) or colonization events (closed bars) were counted. Colonization events corresponded to zones where AM fungi had formed a hyphopodium and penetrated the root (with or without formation of arbuscules). Several combinations of low P (LP) and high P (HP) applied to spores, recipient plants and fertilization during contact were tested (numbered from 1 to 6). Error bars represent the SEM. *n* = 6 plants per combination. Numbers of colonization events were compared across combinations using one-way ANOVA followed by Fisher’s LSD test. Normality of residues was verified using the Kolmogorov–Smirnov’s test. Different letters indicate statistically significant differences (*P* < 0.05).

The marked reduction in mycorrhizal colonization of roots under high P could be attributed to the effects of P on either the plant or the fungus or both plant and fungus. One of the aims of our study was to discriminate between these possibilities. In experiments described above (**Figure 1**), the fungus and plant were grown in the same nutrient solution. We designed an experimental system in which the plant and fungus are grown separately during the pre-inoculation stages of the experiment, and can thus be exposed to different Pi concentrations (see Section “Materials and Methods”). This system was inspired by a synchronized mycorrhization device described by Lopez-Meyer and Harrison (2006). We modified the system to suit our needs (**Figure 4**). Fungal spores were treated *in vitro* with root exudates obtained from plants grown under low P or high P (**Figure 4A**). It is worth noting that spores were exposed to both the nutrient solution itself and to the root exudates produced in this particular nutrient solution. Another set of plants were grown, also under low P or high P (**Figure 4B**), and put in contact with stimulated spores (**Figure 4C**). During co-culture, plants and spores were watered with either low P or high P. This experimental design uncouples the effects of P on the spores (either directly or through the composition of root exudates) from those exerted on the root itself. Various combinations using spores and plants associated with low P or high P fertilization were performed. Hyphopodia and colonization events were counted following co-culture and these results are presented in **Figure 4D**.

Very few colonization events were observed when both plants and spores were treated with high P, as compared with a combination of plants and spores treated with low P (compare combinations 1 and 2 in **Figure 4D**). Combinations of plants and spores treated in different conditions were next examined. A one-way ANOVA identified two groups of combinations: one with high frequency of colonization contained all combinations involving low P recipient plants, and the other with lower frequency of colonization comprised combinations with high P recipient plants. When spores pre-stimulated with low P root exudates were used to inoculate plants pre-grown in high P, irrespective of the fertilization solution used during contact (**Figure 4D**, combinations 3 and 6), very few colonization events were observed. This suggests that the unfavorable conditions applied to plants were dominant to the favorable conditions applied to spores. Conversely, when plants grown under low P were inoculated with spores treated with high P root exudates, roots were colonized to a high level, similar to that observed when both partners were under low P conditions (Figure **4D**, combination 4 vs 1). Therefore, the conditions in which the spores were stimulated again appeared to have little importance. In addition, the nutrient solution used during the contact phase seemed to have little influence on the symbiotic outcome, as shown with the low P spores/low P plants and low P spores/high P plant combinations: colonization levels were not affected by the nutrient solution used during contact (combinations 1 vs**5 and 3 vs 6, respectively, in **Figure 4D**).

### P EFFECTS ON NUCLEAR CALCIUM SPIKING RESPONSES IN THE *M. truncatula* ROOT EPIDERMIS

Under high P conditions the interaction between roots and AM fungi leads to a very low level of colonization. Importantly, the interaction appears to be arrested prior to the formation of hyphopodia (**Figure 4D**), suggesting that very early events are perturbed in these conditions. Among several possibilities, we considered the hypothesis that plants grown under high P might be unable to recognize molecular signals produced by their fungal partner. Since the activation of nuclear-associated calcium oscillations (spiking) is one of the earliest cellular responses to the presence of the fungus, we used this as a marker for the early perception of the fungus by the plant. Spiking analyses can be carried out using *in vitro* root organ cultures derived from *Agrobacterium rhizogenes*-transformed “hairy roots,” since these are particularly well adapted for the observation of the early stages of mycorrhization (Chabaud et al., 2002).

**FIGURE 5:**
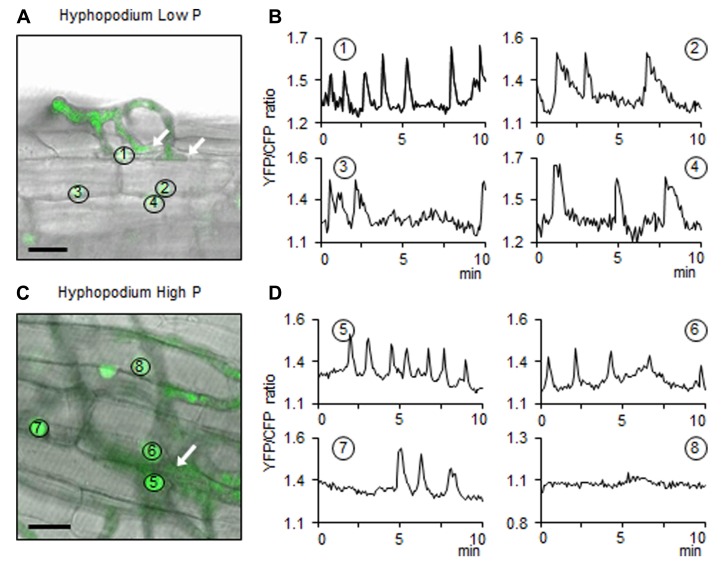
**Nuclear calcium spiking in root cells localized under hyphopodia**. **(A,C)** Hyphopodia (white arrows) formed on low P or high P roots, respectively. Scale bars: 20 μm. The false green color corresponds to the fluorescence produced either by root nuclei containing the cameleon probe or by fungal autofluorescence. Images correspond to the superposition of the bright field image and the fluorescent image. Image **(A)** shows the surface of the root and the contact between a hyphopodium and epidermal cells. Panel **(C)** corresponds to a focal plane underneath the hyphopodium, in the root epidermis. Circles with numbers identify the nuclei in which calcium spiking was recorded. **(B,D)** The graphs represent oscillations of nuclear calcium concentration measured over 10 min in the nuclei shown in **(A)** and **(C)**, respectively. Vertical axis: arbitrary unit for YFP fluorescence/CFP fluorescence ratio, horizontal axis: time (minutes).

The most intense calcium spiking responses have been observed in epidermal cells in response to hyphopodium formation on the root surface (Chabaud et al., 2011). Transgenic root organ cultures of *M. truncatula* expressing the cameleon Nup-YC2.1 nuclear calcium reporter (Chabaud et al., 2011) were used to monitor changes in calcium concentration in epidermal root cells in contact with AM hyphopodia. Roots grown in low P or high P were inoculated with spores of *G. gigantea*. This particular AM fungus was chosen because it possesses naturally fluorescent hyphae which facilitate the observation of hyphal branching and hyphopodia formation (Séjalon-Delmas et al., 1998). Regardless of the P concentration, *G. gigantea* spores germinated equally well and germinating hyphae produced from first- to fourth-order branching. A more intense branching pattern was observed in the immediate vicinity of roots under low P, and only extremely rarely under high P conditions. Likewise, hyphopodia formation was only very rare under high P conditions: when equivalent amounts of roots taken from zones with intense hyphal branching were sampled from low P and high P plates and stained for observation, only two hyphopodia could be observed under high P vs 13 under low P. These results are fully consistent with the mycorrhizal phenotype observed in whole plants (**Figure 4D**). Calcium spiking was monitored in epidermal cells situated directly underneath hyphopodia (**Figure 5**). Under low P (**Figure 5A**), the cells closest to the hyphopodia exhibited calcium spiking of high frequency (**Figure 5B**, nucleus number 1), while spikes were less frequent in cells situated further away from the hyphopodium (**Figure 5B**, nucleus numbers 2, 3, and 4) as previously observed by Chabaud et al. (2011). Under high P, although only rare hyphopodia were formed, similar nuclear calcium spiking was observed in the underlying cells (**Figures 5C,D**). Therefore, once a hyphopodium had formed, the root response in terms of calcium spiking could not be distinguished between low P and high P.

**FIGURE 6:**
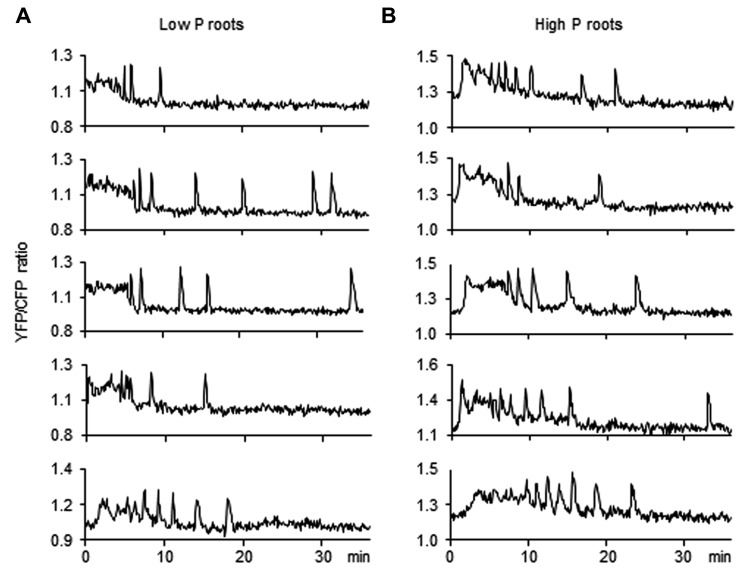
**Nuclear calcium responses induced by GSE treatment in low P and high P roots**. Root organ cultures expressing the nuclear cameleon probe were grown in low P **(A)** or high P **(B)**. Lateral roots were treated with 100 μL of germinated spore exudates (GSEs). The graphs represent calcium oscillations measured in several representative nuclei for each condition. Vertical axis: arbitrary unit for YFP fluorescence/CFP fluorescence ratio, horizontal axis: time (minutes).

In addition to hyphopodium-induced calcium responses, it has also been shown that calcium spiking can be induced by both crude fungal exudates and candidate fungal signal molecules such as Myc-COs (Genre et al., 2013). To investigate whether such responses are perturbed under high P conditions, roots were treated with fungal compounds in the absence of the fungus. In the present study we tested both crude GSEs (Chabaud et al., 2011) and purified chitin tetramers (CO4; Genre et al., 2013). GSEs contain both Myc-LCOs (Maillet et al., 2011) and a variety of short-chain chitooligosaccharides including CO4 (Genre et al., 2013). It is likely that they also contain other biologically active molecules not yet characterized. For the study of calcium spiking responses to fungal compounds, roots were taken either from whole transgenic plants or from transgenic root organ cultures. Studying whole plants takes into account a potential contribution from the P status of the aerial part of the plant. Regardless of the type of root material, the spiking responses were highly irregular over time for a given nucleus, and also quite variable between different nuclei (see **Figures 5 and 6** for root organ cultures). This typical feature of spiking responses to AM fungi makes it difficult to compare response intensities between conditions. Nevertheless, two parameters can be measured unambiguously: the proportion of nuclei that exhibit a spiking response (at least one spike/30 min imaging), and the number of spikes over a given period of time. Although some quantitative aspects of the spiking response may escape this analysis, it certainly allows to determine whether a root responds or not to a given stimulus.

In root organ cultures grown under low P as well as high P, a majority (77–78%) of nuclei exhibited calcium spiking with an average of 3.6–4.4 spikes over 30 min in response to 10^-8^ M CO4 (**Table 2**). Roots of whole transgenic plants also responded strongly to CO4, with cells of low P- and high P-grown plants exhibiting similar calcium spiking responses (83 and 94% of positive nuclei and 6.5 and 4.1 spikes/positive nucleus, respectively; **Table 2**). Finally, the response to *R. irregularis* GSEs was investigated in root organ cultures and once again, roots grown under low P and high P exhibited robust and similar calcium spiking responses (**Table 2**; **Figure 6**). Statistical analyses failed to detect any significant effect of Pi concentration on either the percentage of responding nuclei or the number of spikes over 30 min imaging, whatever the material analyzed and the treatment applied.

**Table 2:** Nuclear calcium spiking in response to CO4 or GSE treatment in low P or high P roots.

Treatment	Plant material	Phosphate condition	Number of analyzed roots	Number of analyzed nuclei	Average proportion of positive nuclei (%)	Average number of spikes/30 min/positive nucleus
10^-^^8^ M CO4	Root organ cultures	Low P	6	56	77	3.6
		High P	8	89	78	4.4
10^-^^8^ M CO4	Whole plants	Low P	4	33	83	6.5
		High P	5	32	94	4.1
GSE	Root organ cultures	Low P	3	27	96	5.5
		High P	3	29	97	5.1

*The table indicates the number of independent root samples analyzed and the number of nuclei in which fluorescence was monitored. Positive nuclei are those showing at least one spike over the 30-min recording period. Data obtained in low P and high P were compared for each combination of treatment and material. No statistically significant difference was found between low P and high P for the percentage of positive nuclei, according to Student’s *t*-test or Mann–Whitney’s test (*P* > 0.9 in all cases). The effect of phosphate concentration on the number of spikes/30 min imaging was tested by nested ANOVA to take into account possible variations between sampled roots (root factor nested in the phosphate concentration). Normality of residues was verified using the Kolmogorov–Smirnov’s test. No statistically significant difference between low P and high P was found using Fisher’s LSD test (*P* > 0.1 for all combinations of treatment and material).*

## DISCUSSION

### CHOICE AND VALIDATION OF P SUPPLY CONDITIONS AFFECTING AM SYMBIOSIS

Although the inhibition of the AM symbiosis by high Pi concentration is a general phenomenon, the concentrations needed to observe this effect depend on the plant species, mode of inoculation and the fertilization conditions. For example, 0.75 mM Pi was sufficient to inhibit the AM symbiosis almost completely in pea (Balzergue et al., 2011), while a concentration of 10 mM Pi was necessary to obtain a similar effect in *Petunia* (Breuillin et al., 2010). In *M. truncatula*, Pi concentrations of 1 and 1.3 mM only had a moderate effect on root colonization (Branscheid et al., 2010; Bonneau et al., 2013, respectively). In our hands, a concentration of 3.75 mM Pi in the nutrient solution almost completely suppressed the AM symbiosis by preventing the formation of hyphopodia. The few hyphopodia that were formed in high P were functional, since they led to normal colonization of the root cortex and the formation of arbuscules (although the overall root colonization was severely reduced due to the limited number of fungal entry points). Therefore, under these experimental conditions, the effects of a high P supply appear to be focused on the earliest stages of the AM association.

The consequences of a high P supply on growth and P accumulation can differ depending on the plant species and culture conditions. In our case, high P conditions resulted in Pi accumulation, especially in leaves, rather than in stimulated growth (**Figure 2**). This small impact of P supply on plant growth is not unusual in *M. truncatula*: similar observations have been reported by other authors on this species (Grunwald et al., 2009; Bonneau et al., 2013). It is possible that under our conditions other nutrients or culture parameters (e.g., light intensity) are more important growth-limiting factors than P. In any case, these results suggest that the responses to P nutrition that depend on internal P concentration rather than on external P availability (Thibaud et al., 2010) should be markedly contrasted between low P and high P. In agreement with this hypothesis, the expression profiles of marker genes of P status under low P and high P (**Figure 3A)** were similar to those described in the literature (Liu et al., 2008; Grunwald et al., 2009; Branscheid et al., 2010). This indicates that after 2 weeks of growth a clear difference in P status is already established between the plants grown under low P or high P.

### HIGH P PRIMARILY AFFECTS THE PLANT PARTNER IN AM INTERACTIONS

Among the mechanisms that could account for the limited root colonization in high P conditions, it is possible to envisage direct effects of external Pi on the fungus (i.e., effects not exerted through the plant via for example a modification of root exudate content). Such effects could include effects on spore germination or hyphal growth, as has been reported in a few cases (de Miranda and Harris, 1994). However, the fact that the Pi concentrations that abolish the symbiosis in pea hardly have any effect in *Medicago* (Balzergue et al., 2011; this manuscript) while an identical fungal inoculum was used, argues against this possibility. Nevertheless, it does not exclude the possibility that these effects become more important at the higher Pi concentrations used for *M. truncatula*. We tested this directly by using the experimental set-up in which plant and fungus are grown separately before contact (**Figure 4**). Germinated spores pre-stimulated under high P were able to successfully colonize low P-grown roots, indicating that the presence of high P during the spore pre-stimulation stage had not decreased their symbiotic capacity.

The same experimental system was exploited to investigate the importance of the potential effects of Pi supply on the composition of root exudates. Phosphate supply has been reported to alter the composition of root exudates, and the amount of inhibitors or activators of AM fungal development (Vierheilig, 2004; Nagahashi and Douds, 2011). These changes could account for the reduced mycorrhizal potential of plants grown in high P (Akiyama et al., 2002; Yoneyama et al., 2007). Such bioactive compounds include strigolactones (Akiyama et al., 2005; Besserer et al., 2006), which on the basis of biosynthetic gene expression (**Figure 3B**) are likely to be less abundant in root exudates produced under high P compared to those produced under low P (Liu et al., 2011a). However, root exudates also contain a number of other compounds potentially active on AM fungi, including various flavonoids (Scervino et al., 2007) and hydroxy fatty acids (Nagahashi and Douds, 2011). The impact of root exudates produced under high P was therefore addressed as a whole, rather than by examining a limited number of known compounds. For this, it is useful to focus on two particular experimental conditions shown in **Figure 4D**: spores pre-stimulated with high P or low P root exudates, both confronted with low P-grown plants (combinations 1 and 4 in **Figure 4D**). In both cases the fungus was able to colonize roots successfully, indicating that root exudates produced in high P did not contain strong inhibitors of AM fungi. Conversely, germinated spores pre-stimulated in low P root exudates poorly colonized high P-grown roots. Therefore, the stimulation of spores with low P root exudates, supposed to be rich in fungal stimulants, was not sufficient to obtain a high rate of root colonization. This is consistent with our previous observation in pea that treatment with exogenous strigolactones was not sufficient to improve root colonization in high P (Balzergue et al., 2011). Furthermore, results in the present article extend this conclusion to the entire, complex content of root exudates.

Finally, only plants grown under low P are efficiently colonized by AM fungi (regardless of the conditions of spore pre-stimulation and fertilization during contact). Thus, the conditions in which the plant has been grown prior to contact determine whether it will be a good or a bad host for AM fungi. Therefore, a high level of P fertilization seems to inhibit AM symbiosis predominantly by acting on the plant itself rather than on the content of its root exudates or on the fungal partner.

### HIGH P DOES NOT INHIBIT THE ROOT CALCIUM SPIKING RESPONSE TO FUNGAL FACTORS

Since mycorrhization under high P was arrested during the earliest stages before fungal attachment to roots, our investigations on possible underlying mechanisms focused on these very early stages. Our data suggest that the presymbiotic plant-to-fungus molecular signaling is affected under high P, but that these effects are not decisive in determining the outcome of the interaction. Alternatively, failure to recognize signals has been proposed as a possible cause of lack of mycorrhizal colonization (Koide and Schreiner, 1992).

To test this further, host nuclear calcium spiking responses to fungal signals were monitored using a cameleon reporter probe (Sieberer et al., 2009). Similar nuclear calcium spiking responses were observed in epidermal cells underneath hyphopodia both under low P and high P (**Figure 5**). In *M. truncatula* mutants defective in genes necessary for the generation (*dmi1*, *dmi2*) or decoding (*dmi3*) of calcium spiking, numerous hyphopodia are formed but the root colonization process is arrested at the root epidermal surface (Marsh and Schultze, 2001). These observations suggest a tight link between calcium spiking and fungal penetration into the roots. Our results are fully consistent with this hypothesis since the few hyphopodia that formed under high P conditions led to apparently normal root colonization events.

Although the root calcium spiking response to hyphopodium formation appears unaffected by P supply, it can be hypothesized that high P decreases the root ability to perceive the fungus prior to contact. This would explain why much fewer hyphopodia are formed under high P conditions. Previous studies have shown that plant roots perceive the presence of AM fungi through diffusible fungal compounds prior to any physical contact (e.g., Kosuta et al., 2003; Mukherjee and Ané, 2011). Notably, calcium spiking in root epidermal cells can be observed in response to fungal exudates or pure compounds such as COs (Chabaud et al., 2011; Genre et al., 2013). Potentially earlier steps in symbiotic communication were therefore examined by analyzing nuclear calcium spiking responses to both crude fungal exudates and potential AM signals. Roots from organ cultures or from whole plants grown under low P and high P responded similarly to both purified chito-tetraose (CO4) and crude GSEs, indicating that these roots can perceive the presence of the fungus at a distance (**Table 2**; **Figure 6**). Roots taken from whole plants grown under low P or high P also displayed a similar calcium spiking response to CO4 (**Table 2**), suggesting that the presence of the aerial part (which is the main site of P accumulation under high P) did not influence the capacity of the roots to respond to these molecules present in fungal exudates. Thus, the inhibition of hyphopodia formation must be explained by some alternative mechanisms, yet to be discovered.

### ALTERNATIVE HYPOTHESES

Various mechanisms could account for the reduced attachment of AM fungal hyphae to high P-grown roots. Observations reported in the literature draw attention to two particular possibilities: the modification by P of recognition patterns present at the root epidermal surface, and a putative hormonal effect of strigolactones on the roots themselves. Several lines of evidence indicate that AM fungi are able to recognize physical patterns on the root epidermal surfaces on which they develop hyphopodia. Firstly, AM fungi can form hyphopodia on cell wall fragments from epidermal cells but not on fragments from other cell types (Nagahashi and Douds, 1997). Secondly, hyphopodia are preferentially formed on grooves between adjacent epidermal cells. Cell walls in these regions are thinner, looser, and richer in unesterified pectin (Bonfante et al., 2000). Thirdly, a glycerol-3-phosphate acyl transferase, involved in cutin and suberin synthesis, was recently shown to be necessary for the formation of hyphopodia on *M. truncatula* roots (Wang et al., 2012). Taken together, these observations point toward an important role of the cell wall composition, and possibly also surface topography, as a hyphopodium differentiation signal for AM fungi. This hypothesis is consistent with studies of appressorium formation in fungal pathogens, which showed the requirement for specific epidermal surface patterns (e.g., Liu et al., 2011b). Although the effects of P supply on epidermal root cell wall composition have not been studied in detail, Pi starvation is known to enhance root cellulose content (Zhang et al., 2012), and to affect the expression of many genes involved in cell wall loosening and biosynthesis (Miura et al., 2011). Therefore, high P could act through modifications of the physical or biochemical properties of the root epidermal surface.

Finally, high P may affect root physiology by altering the hormonal balance. Strigolactones are now recognized as plant hormones involved in several aspects of shoot and root development (Ruyter-Spira et al., 2013), and one of the most striking effects of P supply on hormones is a dramatic reduction of root strigolactone synthesis (Yoneyama et al., 2007; Balzergue et al., 2011; Liu et al., 2011a). On the basis of gene expression data (**Figure 3**), this is most likely the case in the *M. truncatula* plants used in the present study. This raises the question of whether modifications of strigolactone content may affect hyphopodia formation through hormonal effects *in planta*, in addition to the effects of root-exuded strigolactones on the fungus. Strigolactones are known to influence auxin synthesis and transport, thereby modifying root system architecture and root apical meristem function (Ruyter-Spira et al., 2011; Koltai and Kapulnik, 2013). It is likely that other effects of strigolactones on root physiology remain to be discovered. Their role as hormones in mycorrhizal interactions has been investigated by using strigolactone-insensitive mutants, which make it possible to specifically address the role of strigolactones in the plant itself rather than on the fungus. Foo et al. (2013) showed that a strigolactone-insensitive pea mutant is poorly colonized by AM fungi, pointing toward a role for strigolactones *in planta* in the AM association. However, the mycorrhizal symbiosis is further downregulated in high P in this mutant. This observation suggests that reduced strigolactone content is not the only cause of restricted root colonization by AM fungi in high P-grown plants, but does not exclude a contribution of strigolactones. Surprisingly, the analysis of two strigolactone-insensitive mutants of rice affected in different genes gave conflicting results: mycorrhizal root colonization was enhanced in one of the mutants and reduced in the other (Yoshida et al., 2012). Therefore, the hormonal contribution of strigolactone signaling to the symbiosis is not fully understood. Also, strigolactone-insensitive mutants accumulate high concentrations of strigolactones (Umehara et al., 2008; Arite et al., 2009) that are likely to affect the metabolism and transport of other phytohormones (Vanstraelen and Benková, 2012). This makes it difficult to determine whether a particular phenotype is primarily due to strigolactone insensitivity or to perturbations on other hormonal pathways. A detailed analysis of the hormonal implications of P status should help to understand not only how it controls the AM symbiosis, but also how this particular effect is integrated with the other responses to P supply.

## CONCLUSION

Using an experimental system that allowed the application of different P concentrations to the plant and fungal partners, we have shown that high P conditions that inhibit mycorrhizal colonization primarily affect the host roots. In contrast with mutants affected in known elements of the symbiotic pathway, roots grown in high P remain able to respond to fungal signals both at a distance and following contact. This indicates that these roots are not blind to their symbionts, but are unable to interact with them for another, unknown reason. The experimental conditions described in this article should be valuable tools to further investigate these novel regulatory mechanisms in AM symbiosis.

## Conflict of Interest Statement

The authors declare that the research was conducted in the absence of any commercial or financial relationships that could be construed as a potential conflict of interest.

## ABBREVIATIONS

AM: Arbuscular mycorrhiza
CFP: cyan fluorescent protein
CO4: tetramer of *N*-acetylglucosamine
COs: chitooligosaccharides
GSE: germinated spore exudates
LCO: lipochitooligosaccharides
P: phosphorus
Pi: inorganic phosphate
SEM: standard error of the mean
YFP: yellow fluorescent protein
